# SAM-AMP lyases in type III CRISPR defence

**DOI:** 10.1093/nar/gkaf655

**Published:** 2025-07-12

**Authors:** Haotian Chi, Stephen McMahon, Lukas Daniel-Pedersen, Shirley Graham, Tracey M Gloster, Malcolm F White

**Affiliations:** School of Biology, University of St Andrews, St Andrews, Fife KY16 9ST, United Kingdom; School of Biology, University of St Andrews, St Andrews, Fife KY16 9ST, United Kingdom; School of Biology, University of St Andrews, St Andrews, Fife KY16 9ST, United Kingdom; School of Biology, University of St Andrews, St Andrews, Fife KY16 9ST, United Kingdom; School of Biology, University of St Andrews, St Andrews, Fife KY16 9ST, United Kingdom; School of Biology, University of St Andrews, St Andrews, Fife KY16 9ST, United Kingdom

## Abstract

Type III CRISPR systems detect non-self RNA and activate the enzymatic Cas10 subunit, which generates nucleotide second messengers for activation of ancillary effectors. Although most signal via cyclic oligoadenylate, an alternative class of signalling molecule SAM-AMP, formed by conjugating ATP and S-adenosylmethionine, was described recently. SAM-AMP activates a trans-membrane effector of the CorA magnesium transporter family to provide anti-phage defence. Intriguingly, immunity also requires SAM-AMP degradation by means of a specialized CRISPR-encoded NrN family phosphodiesterase in *Bacteroides fragilis*. In *Clostridium botulinum*, the *nrn* gene is replaced by a gene encoding a SAM-AMP lyase. Here, we investigate the structure and activity of *C. botulinum* SAM-AMP lyase, which can substitute for the *nrn* gene to provide CorA-mediated immunity in *Escherichia coli*. The structure of SAM-AMP lyase bound to its reaction product 5′-methylthioadenosine-AMP reveals key details of substrate binding and turnover by this PII superfamily protein. Bioinformatic analysis revealed a phage-encoded SAM-AMP lyase that degrades SAM-AMP efficiently *in vitro*, consistent with an anti-CRISPR function.

## Graphical Abstract

**Graphical Abstract:**
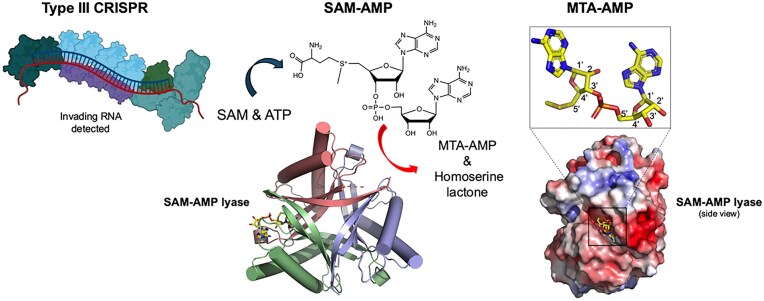

## Introduction

CRISPR–Cas systems function as an adaptive immune system in bacteria and archaea against mobile genetic elements (MGEs) such as viruses and plasmids. Type III systems bind RNA from MGEs, resulting in the activation of the enzymatic Cas10 subunit, which typically comprises a specialized polymerase activity that generates a family of cOA (cyclic oligoadenylate) signalling molecules from ATP [1, 2]. cOA acts as a second messenger of infection in the cell, activating a wide range of ancillary effectors to provide an immune defence. These include nucleases such as Csm6 [2], Csx1 [3], Can1 [4], Can2 [5, 6], and NucC [7, 8] (reviewed in [9]), proteases such as CalpL [10] and SAVED-CHAT [11], transcriptional regulators such as Csa3 [12], translational inhibitors such as Cami1 [13], and transmembrane effectors such as Csx23 and Cam1 [14, 15]. Recently, effector distribution has been analysed and new effector families proposed [16]. Enzymes known as ring nucleases, which can be CRISPR- or virally encoded, degrade cOA to deactivate type III CRISPR systems [17–21].

Recently, a new class of signal molecule, SAM-AMP, has been identified in a *Bacteroides fragilis* type III-B CRISPR system [22]. Two accessory proteins, a CorA family transmembrane protein and an NrN-family phosphodiesterase, were shown to be essential for CRISPR-mediated immunity in the heterologous host *Escherichia coli*. SAM-AMP binding to CorA is thought to potentiate disruption of membrane integrity, resulting in growth arrest or death of infected cells. *In vitro*, NrN functions analogously to ring nucleases, degrading SAM-AMP into SAM and AMP. While this might be expected to represent a means to switch off the immune response, plasmid challenge assays in *E. coli* suggests that NrN activity is required for immunity—an unexpected observation that is still not fully understood [22]. NrN is replaced by a SAM-AMP lyase in some CorA-associated type III CRISPR systems, including that from *Clostridium botulinum* [22]. Previously, phage-encoded SAM lyases have been found to degrade host SAM into 5′-methylthioadenosine (MTA) and l-homoserine lactone (HL), thus neutralizing the bacterial restriction–modification (RM) [23] or bacterial exclusion BREX [24] systems. The phage SAM lyase exhibits structural features characteristic of PII-like signalling proteins: three ferredoxin-like folds assembled into a trimeric structure with a triangular core of β-sheets [23, 25]. Proteins containing this structural feature have the capability to bind ligands like ATP, c-di-AMP, and MTA at the trimeric interface, enabling them to be involved in various cellular processes, including the regulation of anabolic metabolism, metal homeostasis, and SAM degradation [23, 25].

Here, we investigate *C. botulinum* SAM-AMP lyase both *in vivo* and *in vitro*, demonstrating that it is functional in the context of *B. fragilis* CorA-associated CRISPR systems in *E. coli* and specifically degrades SAM-AMP to HL and MTA-AMP. The crystal structure of *C. botulinum* lyase bound to its MTA-AMP product provides insights into SAM-AMP recognition and turnover, as well as providing the first definitive view of a SAM-AMP-derived molecule, which confirms the predicted 5′–3′ phosphodiester linkage. We go on to demonstrate that phage also encode SAM-AMP lyases, which may function as anti-CRISPRs (Acrs) against SAM-AMP signalling defence systems.

## Materials and methods

### Cloning

Cloning of the gene encoding SAM-AMP lyase from *C. botulinum* has been described previously [22]. Briefly, a synthetic gene encoding SAM-AMP lyase was ordered as a g-block (IDT) and cloned between the NcoI and BamHI restriction sites of vector pEhisV5TEV [26] for protein purification, or between the restriction sites NcoI and EcoRI of vector pRATDuet [21] or plasmid pRATDuet-BfrCorA containing *cora* between restriction sites NdeI and XhoI for plasmid challenge assays. Mutations were introduced by site-directed mutagenesis (Phusion High-Fidelity DNA Polymerase, Fisher Scientific) using primers listed in Supplementary Table S1.

### Expression and purification of SAM-AMP lyase

The pEhisV5TEV expression plasmid was transformed into *E. coli* C43 (DE3) cells and grown at 16°C for 18 h after induction with 0.2 mM IPTG (isopropyl β-d-1-thiogalactopyranoside) at an OD_600_ of 0.6–0.8. His-tagged SAM-AMP lyase protein was initially purified by immobilized affinity chromatography [IMAC, 5 ml HisTrap FF column (GE Healthcare)] with gradient elution by increasing the concentration of imidazole from 20 to 500 mM in 50 mM Tris–HCl (pH 8.0), 0.5 M NaCl, and 10% glycerol. The untagged protein was recovered by a second IMAC step, after incubation with TEV protease (1 mg TEV protease per 10 mg target protein) at room temperature during overnight dialysis. Size exclusion chromatography (SEC, HiLoad^®^ 16/600 Superdex^®^ 200 prep grade, Cytiva) was conducted for the final purification step in 20 mM Tris–HCl (pH 8.0), 250 mM NaCl, 10% glycerol, and 1 mM DTT. The homogeneity of SAM-AMP lyase was confirmed by sodium dodecyl sulphate–polyacrylamide gel electrophoresis (SDS–PAGE) (NuPAGE Bis-Tris Gel, Thermo Fisher Scientific), followed by flash freezing, and storage at −70°C.

### Plasmid challenge assay

The method was described previously [22]. Briefly, *E. coli* BL21Star cells (Invitrogen) were co-transformed with both pBfrCmr1-6 and pBfrCRISPR_Tet (or pBfrCRISPR_pUC). A single transformed colony was selected for competent cell preparation. Cells were cultivated in LB medium plus antibiotics (100 μg/ml ampicillin and 50 μg/ml spectinomycin) at 37°C overnight, before 50-fold dilution of the overnight culture into 20 ml LB with the same antibiotics. After OD_600_ reached 0.8–1.0, cell pellets were collected, and resuspended in an equal volume of pre-chilled buffer (60 mM CaCl_2_, 25 mM MES, pH 5.8, 5 mM MgCl_2_, 5 mM MnCl_2_). Solutions were incubated on ice for 1 h and subsequently pelleted, before resuspending in 0.1 volume of the same buffer containing 10% glycerol. Competent cells (100 μl) were transformed with 50 ng pRATDuet, pRATDuet-SAM-AMP lyase, pRATDuet-BfrCorA-SAM-AMP lyase, or SAM-AMP lyase variants. The transformation mixture was incubated at 37°C for 2.5 h with the addition of 0.5 ml LB medium, after heat shock at 42°C for 30 s. A 10-fold serial dilution was applied in duplicate to LB agar plates (supplemented with 100 μg/ml ampicillin and 50 μg/ml spectinomycin) for the determination of the quality of competent cells. The transformants were selected on LB agar containing the same two antibiotics used previously, along with 12.5 μg/ml tetracycline. The full induction was performed in the presence of three antibiotics for selection plus 0.2% (w/v) lactose and 0.2% (w/v) l-arabinose. Plates were incubated at 37°C overnight and imaged. The experiment was performed as two independent experiments with two biological replicates and at least two technical replicates.

### Phage lyase prediction, cloning, expression, and purification

Phage-encoded SAM-AMP lyase candidates were identified based on sequence similarity to the *C. botulinum* protein using BLAST searches. The selected candidate protein (NCBI accession DAN18478.1) is encoded by a *Caudoviricete*sp. phage, identified in the HMP Core Microbiome Sampling Protocol (HMP-A) study of the human viral metagenome [27]. Two further close homologues (DAD71688 and DAT17917) were also detected in the dataset.

A codon-optimized synthetic g-block (IDT) encoding DAN18478 was designed and cloned between the NcoI and BamHI restriction sites of vector pEhisV5TEV [26] for protein expression. The protein was expressed and purified as described for the *C. botulinum* SAM-AMP lyase, with the addition of a final heparin column (Cytiva) chromatography step, where protein was eluted with a gradient of 10 mM to 1 M NaCl in 20 mM Tris–HCl (pH 7.5) and 10% glycerol. The purity and identity of the target protein were confirmed by SDS–PAGE and mass spectrometry.

### SEC analysis

To examine the oligomerization state of *C. botulinum* SAM-AMP lyase wild type (WT) and variants, SEC analysis was performed using a Superose 6 Increase 10/300 chromatography column (Cytiva). The column was equilibrated with SEC buffer containing 20 mM Tris–HCl (pH 8.0), 250 mM NaCl, 10% glycerol, and 1 mM DTT, with a flow rate of 0.5 ml/min. Protein samples were diluted with the same buffer to a concentration of ∼1 mg/ml and 100 μl was injected onto the SEC column. The SEC standards (Bio-Rad) were run under the same conditions, consisting of a mixture of molecular weight markers: thyroglobulin (670 000 Da), γ-globulin (158 000 Da), ovalbumin (44 000 Da), myoglobin (17 000 Da), and vitamin B12 (1350 Da).

### Generation and purification of SAM-AMP

SAM-AMP was purified for crystallographic experiments using *in vivo* production in *E. coli* followed by high-performance liquid chromatography (HPLC). The *in vivo* production was previously described [22]. Briefly, *E. coli* BL21 Star cells containing three plasmids (pBfrCmr1-6, pCRISPR_Tet, and pRAT-Duet) were fully induced with 0.2% (w/v) d-lactose and 0.2% (w/v) l-arabinose when the OD_600_ of the 20-fold diluted overnight culture reached between 0.4 and 0.6. The cell culture, mixed with four times the volume of cold phosphate-buffered saline, was subjected to centrifugation at 4000 × *g* for 10 min at 4°C. Cells were lysed by resuspending them in a cold solvent mixture (acetonitrile:methanol:water, 2:2:1 by volume), vortexed for 30 s, and stored at –20°C until needed. After centrifugation at 13 000 × *g* for 10 min at 4°C, the supernatant was collected, completely dried by evaporation, and resuspended in water for HPLC purification.

SAM-AMP was subsequently purified using a Shimadzu Prominence HPLC system equipped with an HSS T3 HPLC column (Waters 250 mm × 4.6 mm, particle size 5.0 μM). Five hundred microlitre samples were analysed by gradient elution with solvent A (10 mM ammonium bicarbonate) and solvent B (acetonitrile plus 0.1% TFA) at a flow rate of 0.3 ml/min as follows: 0–20 min, 0%–30% B; 20–20.5 min, 30%–100% B. The oven temperature was set at 40°C and the absorbance was monitored at 260 nm. The peak containing SAM-AMP was collected at a retention time of around 11 min with settings of 5 s width, 10 000 μV/s slope, and 800 000 μV level. The pure collected samples were dried into powder.

### Crystal structure determination of SAM-AMP lyase

Initial crystallization conditions for SAM-AMP lyase were obtained after sparse matrix screening of the protein assessing 384 crystallization conditions on a nanolitre scale. SAM-AMP lyase and mother liquor (ML) were mixed in 1:1 or 2:1 protein to ML ratios, in vapour diffusion sitting drop plates. The plates were sealed and left to equilibrate at 293 K. Diffraction quality crystals of WT and E71Q variant protein crystallized in the same condition. Following optimization of the initial hit, the established conditions for crystallization were 67.6% MPD 0.1 M HEPES (pH 7.35), with a protein concentration of 10 mg\ml. To trap SAM-AMP with the protein, E71Q crystals were soaked with a few grains (∼1 μg) of SAM-AMP in the original crystallization drop for 90 min. Prior to data collection all crystals were cryoprotected with ML containing 25% glycerol before cryo-cooling in liquid nitrogen.

X-ray data from WT and E71Q-soaked crystals were collected at a wavelength of 0.9212 Å, at 100 K, on beamline I04-1 at Diamond Light Source. Data were automatically processed using xia2 [28] with DIALS [29] to 1.64 Å (WT) and 1.56 Å (E71Q) resolution, although CC_1/2_ in the outer bin was <0.5 for both. Consequently, data were truncated at 1.70 Å (WT) and 1.65 Å (E71Q) resolution in AIMLESS [30] prior to structure solution to increase CC_1/2_ above 0.5 in the outer resolution bin for each dataset. WT data were phased using PhaserMR [31] in the CCP4 suite [32] utilizing a model generated by AlphaFold2 [33] implemented in Colab, with initial*B*-factors modelled in Phenix [34]. The data from E71Q crystals soaked with SAM-AMP were phased using the WT as the model in PhaserMR.

Both models were refined in the same manner, via iterative cycles of REFMAC5 [35] with manual model manipulation in COOT [36]. For the ligand-bound structure, electron density for MTA-AMP was clearly visible in the maximum likelihood/*σ*_A_ weighted *F*_obs_–*F*_calc_ electron density map at 3*σ*. The coordinates for MTA-AMP were generated in ChemDraw (Perkin Elmer) and the library was generated using acedrg [37], before fitting of the molecule in COOT. Model quality was monitored throughout using MolProbity [38]. Ramachandran statistics for the WT structure are 97.5% favoured and 0% disallowed, and for E71Q structure 98.4% favoured and 0.14% disallowed. Data and refinement statistics are shown in Supplementary Table S2. The coordinates and data have been deposited in the Protein Data Bank with deposition codes 9GAD and 9GAB. Sequence alignment of lyases was performed using Clustal Omega [39] and displayed using ESPript3 [40].

### SAM-AMP degradation assay

The activity of SAM-AMP lyase WT and variants was determined according to the previously published methods [22]. One micromolar SAM-AMP lyase WT and variants were incubated with 100 μM SAM-AMP, S-adenosylhomocysteine-AMP (SAH-AMP), or Sinefungin-AMP (SFG-AMP) generated by *B. fragilis* type IIIB CRISPR complex [22] in 20 mM Tris–HCl (pH 7.5), 250 mM NaCl, and 0.5 mM ethylenediaminetetraacetic acid (EDTA) at 37°C for the indicated time points. The reaction was stopped by adding 2 equivalents of pre-chilled methanol, vortexed for 30 s, and centrifuged at 13 000 rpm and 4°C for 20 min to remove denatured proteins. The supernatant was dried and resuspended in water before HPLC analysis. The activity of the phage-encoded lyase was assessed using the same approach.

### Analytical high-performance liquid chromatography and mass spectrometry

HPLC and MS analyses were conducted as described in [22]. HPLC was conducted using an UltiMate 3000 UHPLC system (Thermo Fisher scientific) coupled with C18 column (Kinetex EVO 2.1 mm × 50 mm, particle size 2.6 μm) for enzymatic sample analysis. LC–MS was performed on an Eksigent 400 LC system equipped with a Sciex 6600 QTof mass spectrometer in positive mode to identify the products of degradation by SAM-AMP lyase.

### Electrophoretic mobility shift assay

One micromolar α-^32^P-radiolabelled SAH-AMP, SAM-AMP, or SFG-AMP, synthesized using *B. fragilis* Cmr complex as described previously [22], were incubated with varying amounts of purified SAM-AMP lyase (0, 0.1, 0.2, 0.3, 1, 5, and 10 μM) in 12.5 mM Tris–HCl (pH 8.0), 5% glycerol, and 0.5 mM EDTA at 25°C for 15 min. Reaction samples mixed with ficoll loading buffer were analysed on a native polyacrylamide gel [8% (w/v) 19:1 acrylamide: bis-acrylamide], with electrophoresis at 200 V for 2 h at room temperature using 1× TBE running buffer. Phosphor imaging was carried out on a Typhoon FLA 7000 imager (GE Healthcare) with photomultiplier tube setting between 700 and 900.

## Results

### *Clostridium* *botulinum* SAM-AMP lyase is functional *in vivo*

**Figure 1.**
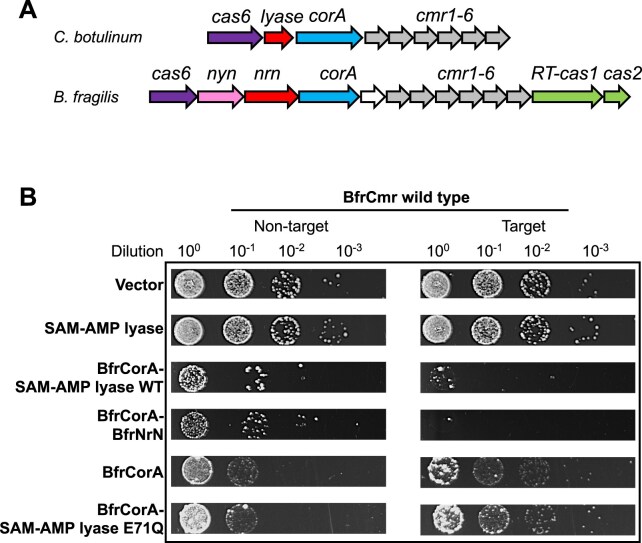
SAM-AMP lyase can replace the NrN phosphodiesterase in a plasmid challenge assay. (**A**) Genome context of the CorA-associated type III-B CRISPR systems in *C. botulinum* and *B. fragilis*. In *C. botulinum*, a predicted SAM-AMP lyase substitutes for NrN in the *B. fragilis* genome, each adjacent to the gene encoding membrane channel protein CorA. The NYN ribonuclease for crRNA processing [49] and the adaption genes cas1 and cas2 in *B. fragilis* are also shown. The type III-B *cas* genes *cmr1–6* and *cas6* are present in both systems. (**B**) Plasmid challenge assay. *Clostridium botulinum* SAM-AMP lyase was tested alone or with *B. fragilis* CorA in the context of *B. fragilis* type III-B CRISPR effector (BfrCmr) programmed with target (tetR) or non-target (pUC19) crRNAs. Cells transformed with a pRATDuet plasmid carrying a tetracycline resistance gene served as a vector control. A pRATDuet plasmid expressing both NrN and CorA served as a positive control for plasmid immunity, which was also observed when NrN was replaced with the *C. botulinum* SAM-AMP lyase. Immunity was lost when the predicted catalytic residue was mutated (E71Q) in SAM-AMP lyase.

Some type III-B CorA-containing CRISPR systems replace the *nrn* phosphodiesterase gene with a gene encoding a predicted lyase (Fig. 1A) and we previously demonstrated that the *C. botulinum* lyase degraded SAM-AMP into 5′-methylthioadenosine MTA-AMP and l-homoserine lactone *in vitro* [22].

Given the strict requirement for both SAM-AMP synthesis and degradation in the *B. fragilis* type III-B CRISPR system [22], we investigated whether SAM-AMP lyase could replace the functional role of the NrN SAM-AMP phosphodiesterase. To assess this, we expressed the *B. fragilis* type III-B system in *E. coli* cells, along with a targeting (pCRISPR-Tet) or non-targeting (pCRISPR-pUC) crRNA. Cells were then challenged by transformation with a pRATDuet plasmid harbouring a *tetR* gene together with genes encoding combinations of CorA, NrN, and SAM-AMP lyase (Fig. 1B and Supplementary Fig. S1). Cells with an active CRISPR system and *tetR* targeting crRNA prevent plasmid transformation [22]. As observed previously, CorA did not prevent plasmid transformation unless the NrN protein was also expressed, although some toxicity was observed even for non-targeting crRNA. High levels of CorA expression also result in smaller colony size, suggestive of a deleterious effect in the absence of the type III CRISPR system (Supplementary Fig. S1).

Expression of SAM-AMP lyase in the context of an active CRISPR system did not affect transformation efficiency, showing the same transformation level as the vector control. However, co-expression of SAM-AMP lyase and CorA resulted in a reduced number of colonies for the activated CRISPR system, mirroring the phenotype observed for NrN (Fig. 1B). Notably, when a SAM-AMP lyase variant with a mutation in the predicted catalytic site (E71Q) was present, colony formation patterns resembled those observed with only CorA expression. These findings confirm the strict requirement for SAM-AMP degradation activity in the CorA-containing system and demonstrate that SAM-AMP lyase functions analogously to the NrN phosphodiesterase in this context, albeit this requirement is still not fully understood.

### Binding and cleavage of SAM-AMP and analogues by SAM-AMP lyase

**Figure 2.**
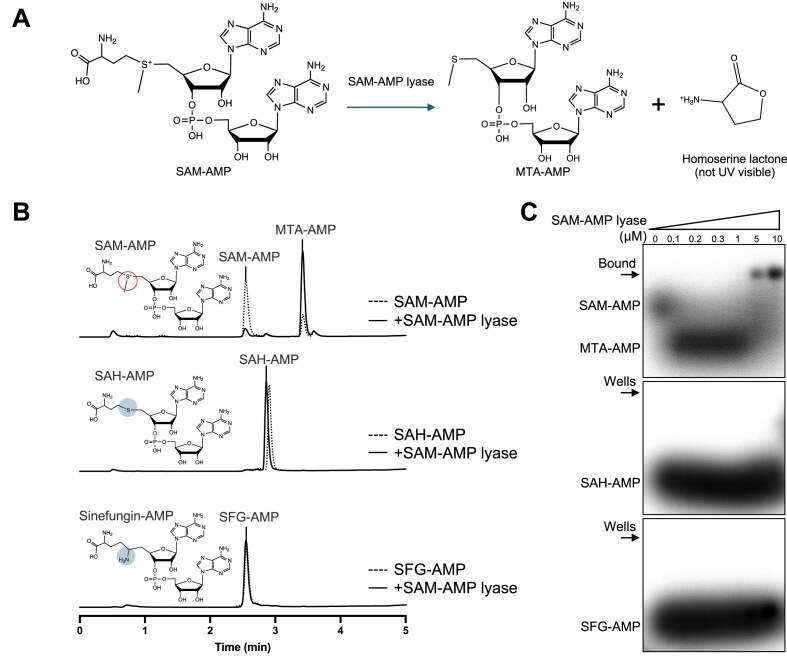
Binding and cleavage of SAM-AMP and analogues. (**A**). Schematic representation of SAM-AMP degradation catalysed by *C. botulinum* SAM-AMP lyase. The product homoserine lactone (HL) is not UV detectable. (**B**). SAM-AMP lyase specifically cleaves SAM-AMP to MTA-AMP and HL. HPLC analysis of reactions in which SAM-AMP lyase was incubated with SAM-AMP, SAH-AMP, or SFG-AMP. Circles highlight their structural difference in the sulphur centre. (**C**). SAM-AMP lyase binds its product MTA-AMP, but not SAH-AMP or SFG-AMP. One micromolar ^32^P-labelled ligands incubated with 0, 0.1, 0.2, 0.3, 1, 5, and 10 μM SAM-AMP lyase were analysed by native gel electrophoresis and imaged by phosphor imaging. Representative figures of three repeats are shown.

To assess the substrate specificity of SAM-AMP lyase (Fig. 2A), we generated unlabelled and ^32^P-radiolabelled versions of SAM-AMP and its analogues SAH-AMP and SFG-AMP, which exhibit structural variability at the sulphur centre of the methionine moiety. HPLC analysis and mass spectrometry confirmed that SAM-AMP was degraded, generating the previously observed MTA-AMP product (Fig. 2B and Supplementary Fig. S2). In contrast, neither SFG-AMP nor SAH-AMP were substrates for SAM-AMP lyase (Fig. 2B), confirming the predicted requirement of the lyase reaction for a positively charged sulphonium ion. SAM-AMP lyase was incubated with the radiolabelled molecules and subsequently analysed via native gel electrophoresis. SAM-AMP lyase was active in the binding buffer, resulting in a shift in the radioactive signal to a faster migrating band corresponding to MTA-AMP (Fig. 2C, top panel). Clear retarded species were observed at higher concentrations of SAM-AMP lyase, which likely represent bound MTA-AMP. In contrast, SAH-AMP and SFG-AMP did not exhibit a shift (Fig. 2C), suggesting that these analogues are not tightly bound by SAM-AMP lyase.

### Trimeric structure of *C. botulinum* SAM-AMP lyase

**Figure 3.**
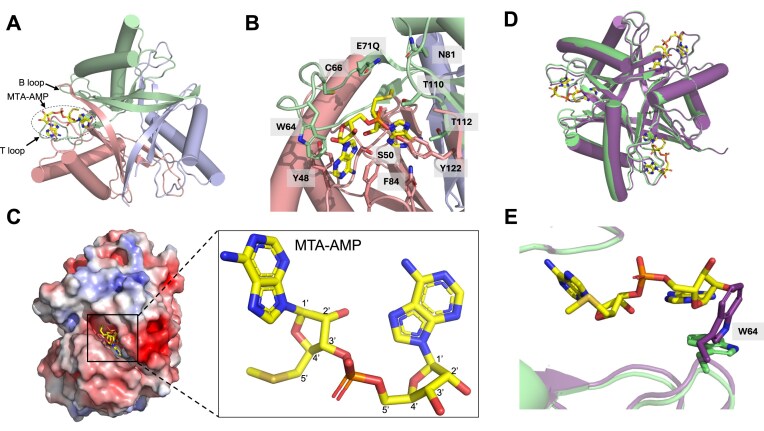
Structure of *C. botulinum* SAM-AMP lyase. (**A**) Trimeric structure of SAM-AMP lyase E71Q variant in complex with MTA-AMP. The three monomers are coloured green, blue, and salmon. Only one MTA-AMP molecule (yellow sticks), bound at the interface between two subunits, is shown. The T-loop and B-loop in proximity to the MTA-AMP are indicated by arrows. (**B**) Binding site for SAM-AMP, with interacting residues shown in the same colours as panel (A). (**C**) Surface representation, coloured by electrostatic potential [blue, (electro)positive; red, (electro)negative; white, non-polar], of SAM-AMP lyase trimer. MTA-AMP molecule is shown as yellow sticks. (**D**) Trimeric structure of SAM-AMP lyase E71Q variant (green) in complex with MTA-AMP (yellow sticks) superimposed onto the trimeric structure of WT apo SAM-AMP lyase (purple). (**E**) Binding site for SAM-AMP, showing change in position of Trp64; colours as shown in panel (D).

Purified WT SAM-AMP lyase (Supplementary Fig. S3) was crystallized, X-ray data were collected to 1.70 Å resolution, and the structure was solved using molecular replacement (Fig. 3). Crystals of the E71Q variant, targeting a key predicted active site residue, were soaked with SAM-AMP prior to vitrification and data collected to 1.65 Å resolution. The asymmetric unit contains six copies of SAM-AMP lyase, with two trimeric complexes. SAM-AMP lyase exhibits a typical trimeric PII-like protein architecture characterized by a triangular core formed by the four-stranded antiparallel β-sheet of each subunit (Fig. 3A). Each monomer possesses a signature ferredoxin fold (β1-α1-β2-β3-α2-β4), featuring a β-sheet on one side and two α-helices on the other. The β2 and β4 strands from each of the three subunits interact in a ‘head-to-tail’ orientation, contributing to most of the trimeric interface. The T-loop connecting β2 and β3, which plays a key role in interactions with target proteins in canonical PII proteins [41, 42], is shorter (10–12 residues in SAM-AMP lyase/SAM lyase compared to >20 residues in homologues) and presumably less flexible in SAM-AMP lyase/SAM lyase (given the loops are fully modelled in the lyases, but missing in the homologues, despite similar data resolution). The B-loop, located between α2 and β4, is another conserved loop in the PII protein superfamily that forms interactions with ligands [42].

### Active site of SAM-AMP lyase

Although SAM-AMP lyase E71Q variant crystals were soaked with SAM-AMP, the product following cleavage, MTA-AMP, was observed in the maximum likelihood/*σ*_A_ weighted *F*_obs_–*F*_calc_ electron density map at 3*σ*, indicating turnover had occurred (Supplementary Fig. S4). MTA-AMP binds at the interface between two monomers in the trimeric complex. Several residues on the T-loop (comprising residues 59–71) and B-loop (comprising residues 106–111) of SAM-AMP lyase constitute the binding site for MTA-AMP (Fig. 3B). The adenine base of AMP is stabilized by π-stacking interactions with the side chains of W64 and F84. T110 and Y122 form hydrogen bonds with the bridging phosphate moiety and the adenine base of MTA interacts with T112 and S50. Other conserved residues Y48, C66, E71, N81, and Q108 (Supplementary Fig. S5) form the rest of the binding site but make no direct interactions with MTA-AMP (Fig. 3B). Note that of the residues discussed, F84, Y122, S50, Y48, and N81 are from a different subunit to the others, demonstrating the significant contributions of neighbouring monomers to ligand binding. The crystal structure of MTA-AMP bound in SAM-AMP lyase confirms the predicted 3′–5′ phosphodiester bond between the SAM and AMP moieties formed by Cas10 [22].

MTA-AMP is buried in a ‘slot-like’, predominantly electronegative, cleft between the subunits of SAM-AMP lyase (Fig. 3C), with the predicted position of the carboxyl group in the substrate SAM-AMP orientated towards the B-loop (Fig. 3A). Comparison of the apo SAM-AMP lyase and in complex with MTA-AMP reveals almost identical structures (root mean square deviation (RMSD) of 0.3 Å over 123 Cα atoms) (Fig. 3D), consistent with the slight conformational change observed in the homologous SAM-lyase (Svi3-3) upon SAM binding [23]. This suggests the proteins essentially have a pre-formed binding site for ligands. Given the proximity of the T-loop to the binding site of MTA-AMP, it is perhaps surprising that there is no significant movement of this upon binding. However, the side chain of W64, which could only be modelled in some monomers suggesting that it is highly flexible, appears to move through 90° from a position that would clash with SAM-AMP to a conformation promoting the π-stacking interaction with the adenine base of the AMP (Fig. 3E). This residue may serve as a gatekeeper to the binding site, locking the SAM-AMP substrate in position for catalysis. Interestingly, however, W64 is not conserved in all SAM-AMP lyases (Supplementary Fig. S5), so the implication of this movement needs further exploration.

### Comparison of SAM-AMP lyase with SAM lyase (Svi3-3)

**Figure 4.**
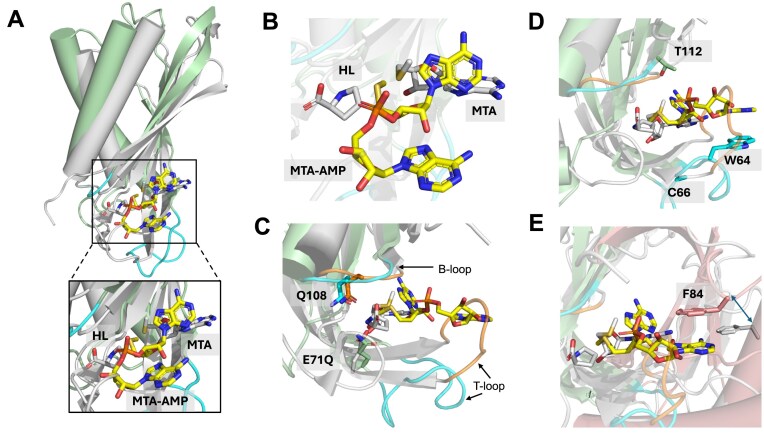
Comparison of structure and active site of SAM-AMP lyase with SAM lyase. (**A**) Comparison of monomer structure of SAM-AMP lyase (green) and SAM lyase (Svi3-3, grey) in complex with their ligands, MTA-AMP (yellow sticks) and MTA (grey sticks), respectively. The B-loop (upper) and T-loop (lower) are shown in cyan for SAM-AMP lyase. (**B**) Comparison of conformation and position of MTA-AMP (yellow) and MTA (grey) in binding site when the SAM-AMP lyase and SAM lyase are superimposed. (**C**) Comparison of active site features for SAM-AMP lyase (green), with the B-loop (upper) and T-loop (lower) shown in cyan in complex with MTA-AMP (yellow), overlapped with SAM lyase (grey), with the B-loop and T-loop shown in orange, in complex with MTA (grey). The only structurally conserved residues between the enzymes are the catalytic residue E71 (shown here as E71Q variant) and Q108. (**D**) Comparison of active site features for SAM-AMP lyase and SAM lyase, coloured as in panel (C), highlighting SAM-AMP residues in the B-loop and T-loop that interact with MTA-AMP, but which are too remote in SAM lyase to form interactions with MTA. (**E**) Comparison of active site residues at the dimer interface features for SAM-AMP lyase and SAM lyase, coloured as in panel (C), with the second monomer shown in salmon for SAM-AMP lyase and grey for SAM lyase. F84, which forms a stacking interaction with the adenine in MTA-AMP, is conserved by sequence with SAM lyase (Supplementary Fig. S3), but is not structurally conserved (indicated by arrow), with the equivalent residue in SAM lyase distant from the active site and unlikely to interact with the ligand. All residue numbering shown corresponds to SAM-AMP lyase.

Superposition of SAM-AMP lyase in complex with MTA-AMP and SAM lyase in complex with MTA (PDB 6ZM9) gave an RMSD of 2.1 Å over 106 Cα atoms (Fig. 4A). Despite the conservation of residues between the two enzymes, there are differences in how the ligands bind (Fig. 4B). The adenosine moiety is rotated by ∼160° in SAM-AMP lyase compared to SAM lyase; while the adenine base and methionine moiety for each protein are roughly in the same location of the binding pocket, the ribose sugars are ∼5 Å apart. The structures show that E71 of SAM-AMP lyase, positioned at the C-terminus of the T-loop, corresponds to E69 in SAM lyase, which is crucial for catalysis (Fig. 4C) [23]. The B-loop (residues 106–111 in SAM-AMP lyase) superimposes well with SAM lyase, with the conserved residue Q108 (SAM-AMP lyase) overlapping with Q104 (SAM lyase) (Fig. 4C). T112 in SAM-AMP lyase, which interacts with the adenine base, however, is not conserved in SAM lyase (which has an isoleucine in equivalent position) (Fig. 4D). The conformation of the T-loop (residues 59–71) differs significantly between SAM-AMP lyase and SAM lyase, meaning residues W64 and C66 in SAM-AMP lyase are not conserved as the equivalent position in SAM lyase is not close enough to MTA to interact directly (Fig. 4D). The difference in position is likely due to the addition of the AMP to SAM-AMP substrate, as the loop in SAM lyase would clash with this group. Like SAM-AMP lyase, the interactions made by SAM lyase come from two adjacent monomers in the trimeric protein. Strikingly, however, of the residues from both monomers of SAM-AMP lyase that interact with MTA-AMP, there is only structural conservation of E71 and Q108 in SAM lyase. F84 is conserved by sequence (Supplementary Fig. S6), but the phenylalanine in SAM lyase is not observed in the same position structurally (Fig. 4E).

### Probing the role of active site residues

**Figure 5.**
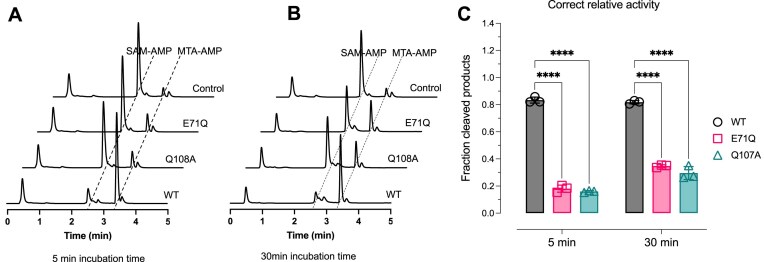
SAM-AMP lyase activity of WT and variant proteins. (**A**) HPLC analysis of SAM-AMP cleavage activity of WT and variants of *C. botulinum* SAM-AMP lyase, in which 1 μM lyase was incubated with 100 μM SAM-AMP for 5 min [or 30 min, (**B**)]. (**C**) The relative SAM-AMP cleavage activity by quantification of the generation of its product MTA-AMP. Data from three independent experiments are shown, with mean ± SD calculated. Statistical significance was calculated using two-way analysis of variance followed by Dunnett’s multiple comparisons test (****P< .0001, GraphPad Prism 9).

Given the conservation of E71 and Q108 in the SAM-AMP lyases and SAM lyase, which are in the T-loop and B-loop, respectively, we set out to understand whether they played a role in substrate binding or catalysis. We also wanted to probe the role played by C66 that is strictly conserved in SAM-AMP lyases. To investigate this, the E71Q, C66A, and Q108A variants of SAM-AMP lyase were expressed and purified (Supplementary Fig. S3). Activity assays revealed that the degradation activity was significantly reduced for the E71Q and Q108A variants, while the C66A variant showed SAM-AMP turnover similar to that of the WT. These results suggest significant roles for E71 and Q108 in catalysis, consistent with their conservation between SAM-AMP lyases and SAM lyases, although neither residue is absolutely essential for activity (Fig. 5A and B, and Supplementary Fig. S7A and B).

### Phage encoded SAM-AMP lyases are potential anti-CRISPRs

**Figure 6.**
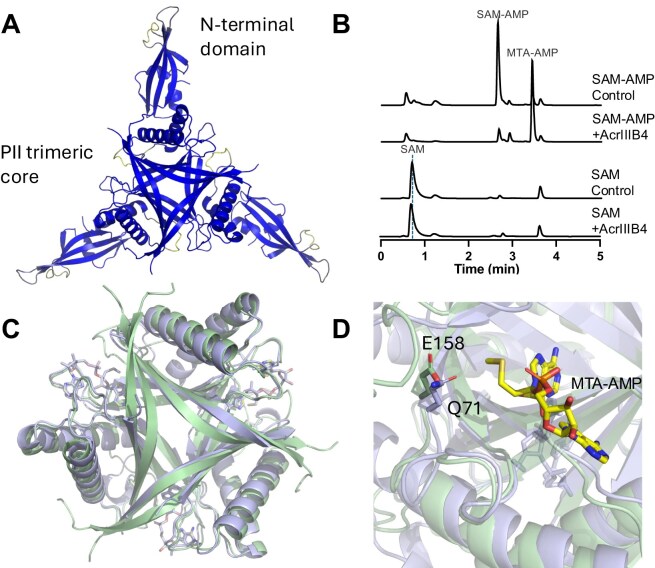
Predicted structure and activity of phage-encoded SAM-AMP lyase. (**A**) A predicted structure model (AlphaFold3 [50]) of the phage SAM-AMP lyase (candidate AcrIIIB4) coloured according to the pLDDT (predicted local distance difference test) [33]. (**B**) Specific SAM-AMP cleavage activity of AcrIIIB4. One micromolar AcrIIIB4 was incubated with 100 μM SAM-AMP or SAM for 60 min, followed by HPLC analysis. The cleavage products were MTA-AMP and HL (not UV detectable). (**C**) Comparison of predicted AcrIIIB4 trimer model (green) and the CboSAM-AMP lyase E71Q variant with MTA-AMP complex (blue). RMSD is 2.1 Å over 112 Cα atoms. (**D**) The ligand binding pocket of AcrIIIB4 (blue) and SAM-AMP lyase (green). The conserved catalytic residues E71 (here mutated to a glutamine) in SAM-AMP lyase and E158 in AcrIIIB4 are shown as sticks. The MTA-AMP reaction product is shown with carbon atoms coloured yellow.

Nucleotide second messengers such as cOA and SAM-AMP are potent activators of type III CRISPR defence. As such, they are attractive targets for virus-encoded anti-CRISPR (Acr) enzymes that degrade these molecules, neutralizing cellular defences (reviewed in [43]). We thus searched for potential phage-encoded SAM-AMP lyases in the NCBI database, identifying a candidate protein (DAN18478) encoded by a phage *Caudoviricetes* species isolate in a viral metagenome dataset isolated from the human microbiome [27]. Two highly similar orthologues (DAD71688 and DAT17917) from the same dataset were also identified. We predict that DAN18478 encodes a trimeric PII-like protein of the SAM lyase family, fused to an N-terminal domain of unknown function (Fig. 6A). The gene encoding DAN18478 was recombinantly expressed in *E. coli* and purified to near-homogeneity, allowing biochemical characterization (Supplementary Fig. S8A). The phage protein was an active SAM-AMP lyase, generating an MTA-AMP product as observed for the bacterial enzyme, while neither SAM nor SAH-AMP or SFG-AMP lyase activity was detected (Fig. 6B and Supplementary Fig. S8B). Furthermore, the phage enzyme could substitute effectively for *C. botulinum* SAM-AMP lyase in plasmid challenge experiments when combined with the *B. fragilis* Cmr/CorA system (Supplementary Fig. S9). Sequence and structural analysis predicted the conservation of a conserved acidic residue (E158) at a position equivalent to E71 of SAM-AMP lyase (Fig. 6C and D, and Supplementary Fig. S3). Unfortunately, a E158Q variant of the phage enzyme could not be purified due to low solubility. We were unable to directly test Acr activity *in vivo*, as the *B. fragilis* type III CRISPR complex requires SAM-AMP degradation for immune function in our heterologous *E. coli* experimental system [22]. With these caveats, we tentatively assign the name AcrIIIB4 (fourth identified Acr of type IIIB systems) for this phage SAM-AMP lyase, but emphasize that this needs confirmation.

## Discussion

The recent discovery [22] that a clade of type III CRISPR systems respond to infection by synthesizing a novel signalling molecule, SAM-AMP, was unexpected. While SAM-AMP binding to a predicted membrane effector of the CorA family is reminiscent of the activation of effectors by cOA species, the observed requirement for SAM-AMP degradation to achieve immunity remains unusual and hard to explain. Although ring nucleases that degrade cOA species are frequently associated with type III CRISPR loci [9, 44], they are not essential for immunity and are thought to function in the resetting of the system once an infection has been cleared [45]. In contrast, there is no immunity (in a reconstituted, heterologous system) without an enzyme that degrades SAM-AMP [22]. Here, we have confirmed that both specialized SAM-AMP NrN phosphodiesterases and lyases can fulfil the same role in the immune response. As these two enzymes generate very different reaction products, this rules out the possibility that a processed form of SAM-AMP is the active signalling molecule. The requirement for this activity remains enigmatic and may require detailed analysis of the mechanism of CorA effector activation and/or studies of cognate host/phage systems.

The structures of SAM-AMP lyase in apo and product-bound forms provide the first detailed view of this enzyme. SAM-AMP lyase is representative of the trimeric PII superfamily structure and closely related to the phage SAM lyase [23] with a key conserved catalytic glutamate residue. For SAM lyase, the suggested mechanism requires dehydration of the carboxylate moiety of SAM in the binding pocket, leading to attack of the sulphonium centre and formation of HL and MTA-AMP in a unimolecular reaction [23]. Although SAM-AMP lyase E71Q variant crystals were soaked with SAM-AMP, the electron density revealed only the bound reaction product MTA-AMP, with no density corresponding to HL in the binding pocket. We postulate that HL is not tightly bound by the enzyme, resulting in its diffusion from the active site, or adoption of a range of conformations in the binding pocket that preclude its identification in the electron density. The MTA moiety is bound in a broadly similar position, albeit with variations in conformation and interactions formed, to that observed in the SAM lyase structure. There is an extended binding site for the AMP moiety, which affords SAM-AMP lyase specificity for SAM-AMP over SAM, which is not a substrate. The accommodation of the AMP, however, means a significant difference in the position of the T-loop in the two enzymes, and in fact only two residues are structurally conserved. The co-crystallization of MTA-AMP provides unambiguous evidence for a 5′–3′ phosphodiester bond linkage, which was previously predicted but could not be proven based on mass spectrometry data alone [22].

The signalling molecules generated by prokaryotic and eukaryotic immune systems represent an attractive target for viral counter-defence measures. Viral ‘ring nucleases’ degrade cA_4_ to neutralize type III CRISPR systems [21], cyclic nucleotides used in CBASS signalling and the cGAMP signalling molecule generated by cGAS in eukaryotic antiviral defence [46–48]. Indeed, phage SAM lyase likely functions by disrupting antiviral defence, albeit by targeting a ‘housekeeping’ cofactor [24]. The discovery of a phage-encoded enzyme specific for degradation of SAM-AMP fits this paradigm. We envisage that sequestration and degradation of the SAM-AMP molecule prevents activation of the CorA effector and neutralization of immunity. Although we designate this phage enzyme as the anti-CRISPR AcrIIIB4, functional confirmation *in vivo* will require the study of a cognate host/phage system. Given that the only known function of SAM-AMP is in antiphage defence and that spacers corresponding to phages encoding the candidate AcrIIIB4 are found in the genomes of *Leptotrichia* species [27], which utilize CorA-mediated CRISPR defence, this prediction is feasible.

In conclusion, our work provides the first view of a protein that binds and turns over the recently discovered SAM-AMP signalling molecule in type III CRISPR defence, providing a framework for future studies.

## Supplementary Material

gkaf655_Supplemental_File

## Data Availability

The protein structure coordinates and data have been deposited in the Protein Data Bank with deposition codes 9GAD and 9GAB.

## Notes

Present address: Department of Life Sciences, Imperial College London, London, SW7 2AZ, United Kingdom
