# Supplementary material for: SAM-AMP lyases in type III CRISPR defence
Source: Nucleic Acids Res. 2025 Jul 12;53(13):gkaf655. doi: 10.1093/nar/gkaf655 (PMC12255297; doi:10.1093/nar/gkaf655)
Supplement: gkaf655_Supplemental_File [file gkaf655_supplemental_file.pdf]

SAM-AMP lyases in type III CRISPR defence

Haotian Chi, Stephen McMahon, Lukas Daniel-Pedersen, Shirley Graham, Tracey M Gloster* and Malcolm F White*

Supplementary Figures

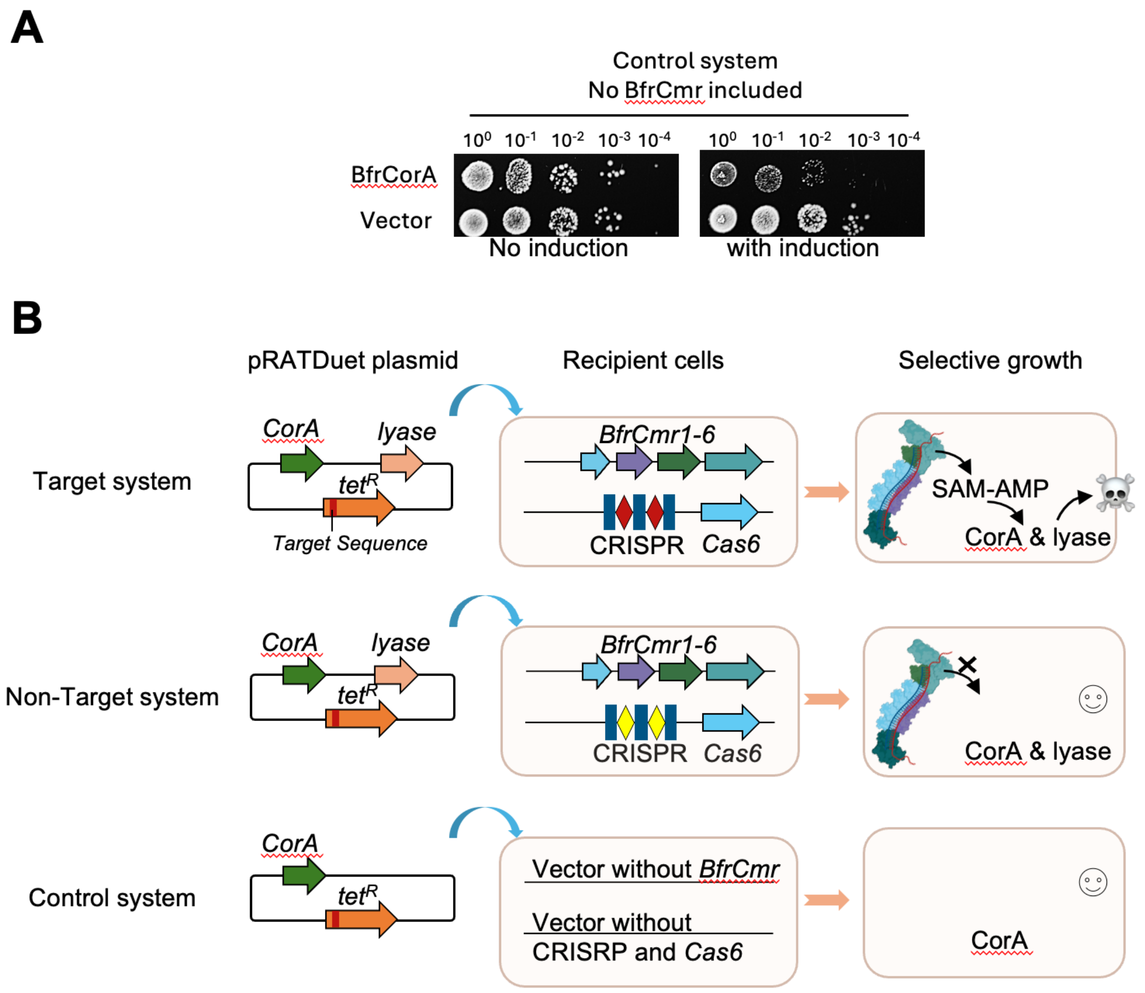

Supplementary Figure 1. Plasmid challenge assays.

A. The vector pRATDuet expressing *B fragilis* CorA or not was tested in the control system, which did not contain *B. fragilis* Cmr system. Transformants were selected in the absence (no induction) or presence (with induction) of arabinose and lactose. **B.** schematic illustrations of the assays.

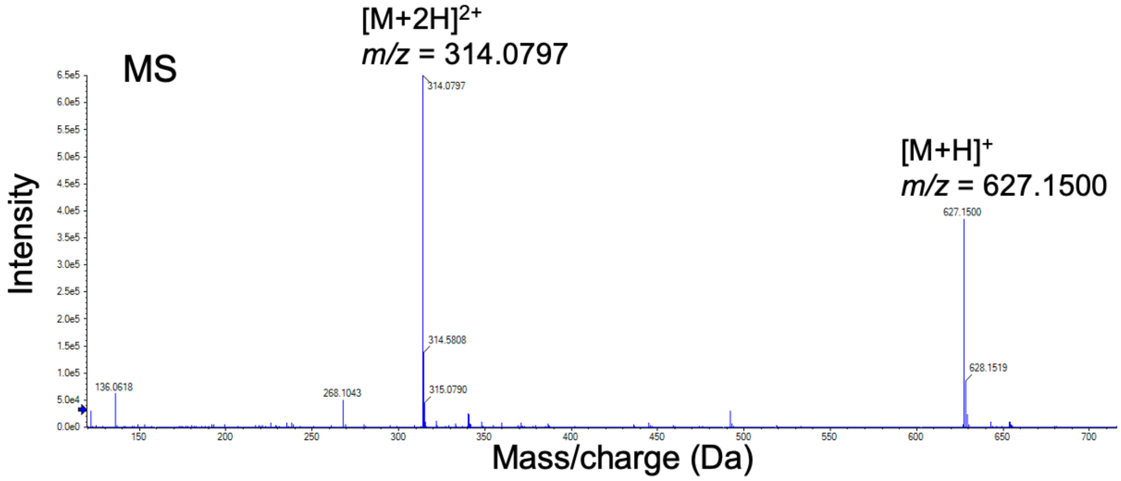

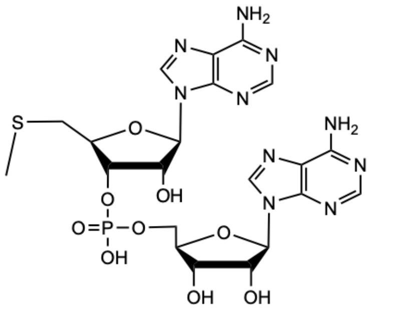

MTA-AMP
Molecular Weight: 626.5418
Theoretical [M+H]$^{+}$ m/z = 627.1494
Theoretical [M+2H]$^{2+}$ m/z = 314.0783

Supplementary Figure 2. Verification of SAM-AMP lyase cleavage products.

Mass spectrometry (top) was conducted in positive mode and detected the cleavage product MTA-AMP in two different ionization modes, [M+H]$^{+}$ and [M+2H]$^{2+}$, consistent with its theoretical m/z (bottom).

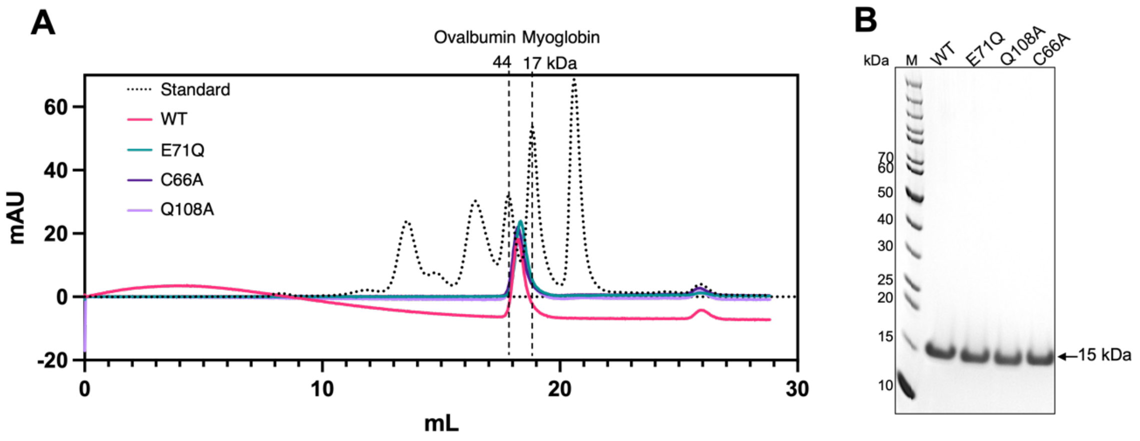

Supplementary Figure 3. Size exclusion chromatography (SEC) of SAM-AMP lyase wild type and variants

A. SEC analysis of SAM-AMP lyase wild type and variants, which all eluted as a trimer (~30 kDa) between the standards for ovalbumin (44 kDa) and myoglobin (17 kDa). **B.** SDS-PAGE analysis of SAM-AMP lyase wild type and variants. The observed mass of SAM-AMP lyase on the gel is about 15 kDa, consistent with its theoretical monomer mass. M is the molecular weight marker with sizes indicated.

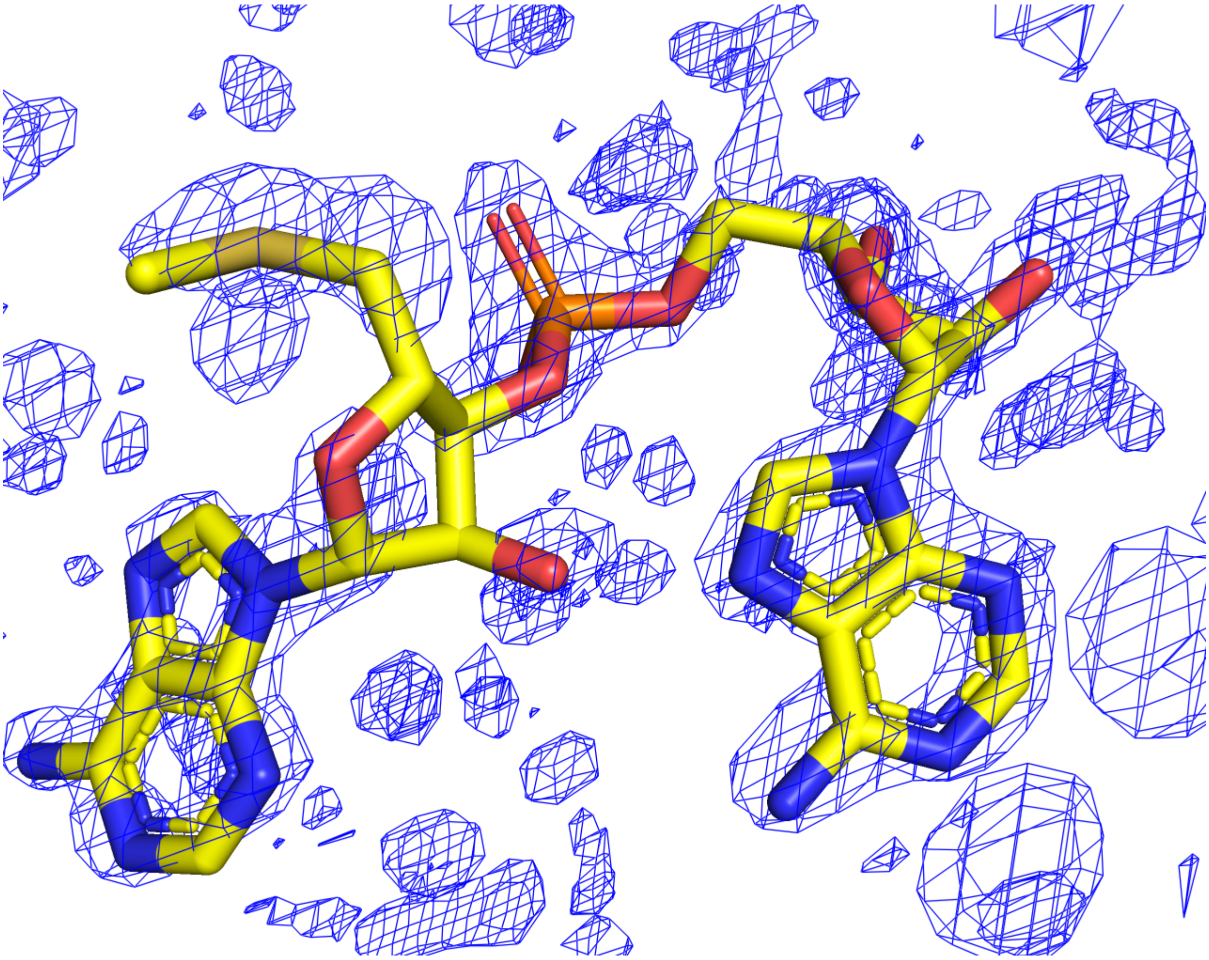

Supplementary Figure 4. OMIT map of the region surrounding MTA-AMP, contoured at 3σ.

The electron density is shown as a blue mesh and was generated with MTA-AMP omitted from the model to produce an unbiased map. This map was calculated prior to modelling MTA-AMP into the structure. For illustration purposes, MTA-AMP atoms have been superimposed, but were not included in the map calculation.

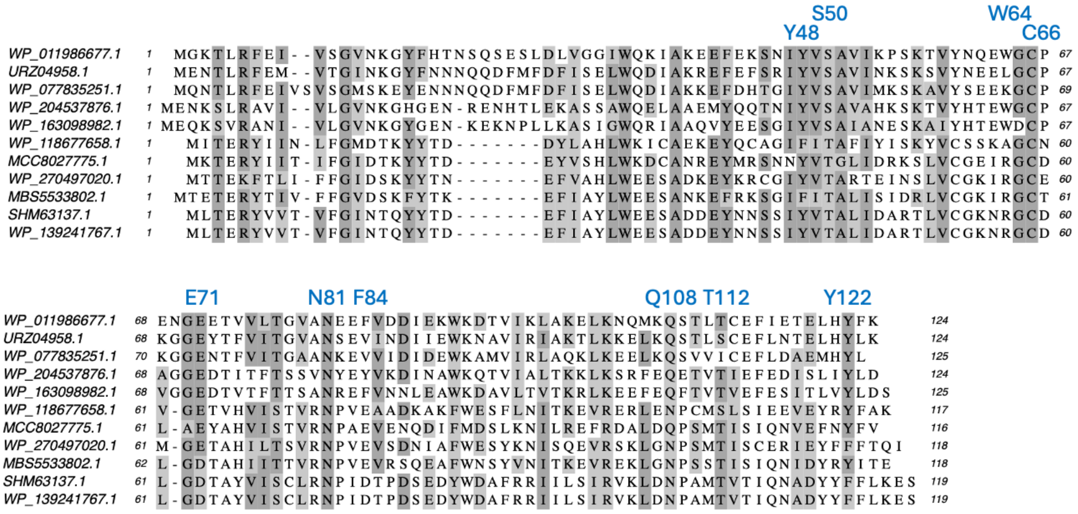

Supplementary figure 5. Multiple sequence alignment of a range of bacterial SAM-AMP lyase homologues.

Residues mentioned in the text are labelled. NCBI accession codes are shown. The top sequence, WP_011986677.1, is the *C. botunlinum* protein.

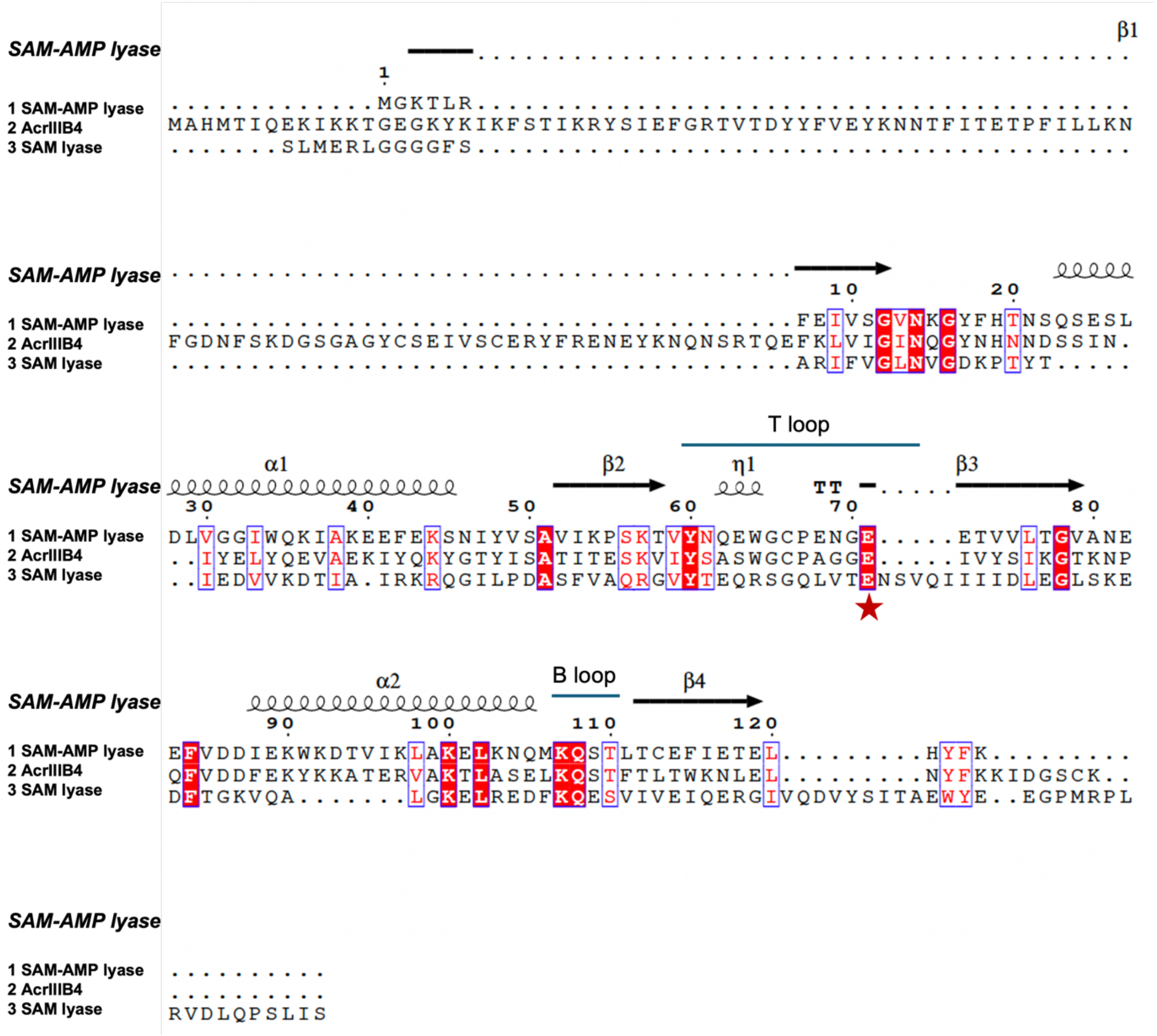

Supplementary Figure 6. Structure-guided sequence alignment of lyases.

Secondary structure of SAM-AMP lyase is displayed above the sequence alignment of SAM-AMP lyase, phage lyase (AcrIIIB4) and SAM lyase (Svi3-3). The white letters on a red background show strictly conserved residues, with the conserved catalytic glutamate residue highlighted as a red star. The red letters with blue frames represent relatively conserved residues. Figure was generated using ESPript3 (40).

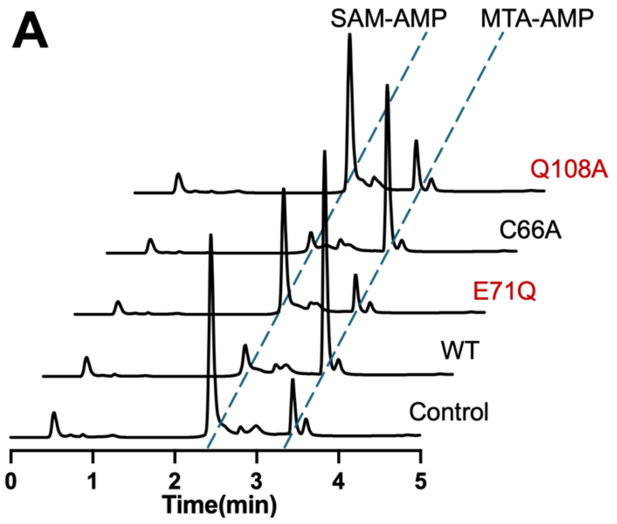

10 min incubation time

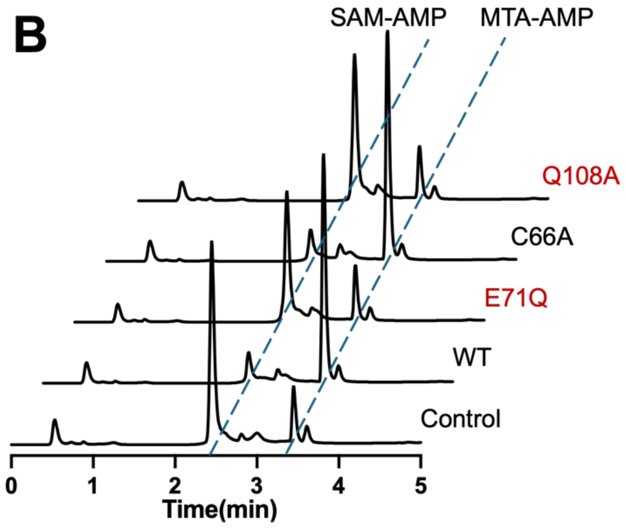

30 min incubation time

Supplementary Figure 7. SAM-AMP cleavage activity of SAM-AMP lyase wild type and variants

A. Comparison of cleavage activity of SAM-AMP lyase wild type and variants. The generation of cleavage product MTA-AMP was assessed by HPLC. C66A showed similar activity as WT, while the degradation activity was significantly reduced for the variants E71Q and Q108A, after incubation with SAM-AMP for 10 min, or 30 min shown in **B**.

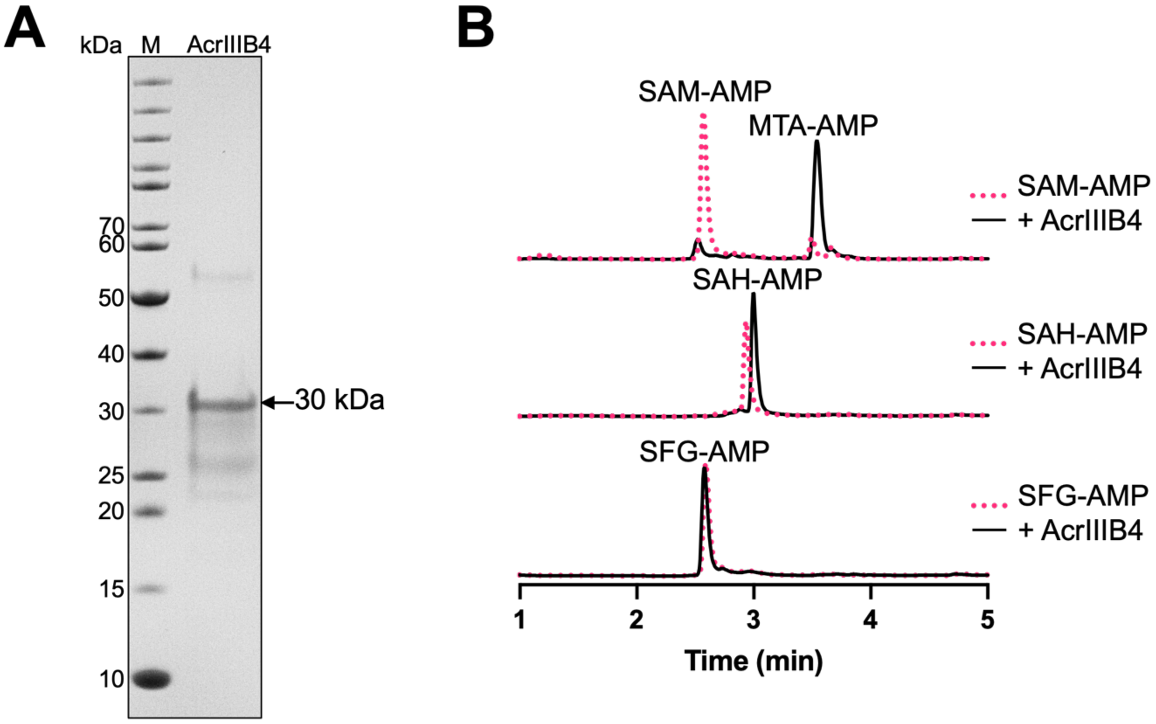

Supplementary Figure 8. Cleavage of SAM-AMP and analogues by phage SAM-AMP lyase.

A. SDS-PAGE analysis following recombinant expression of AcrIIIB4. The observed mass of AcrIIIB4 on the gel is about 30 kDa, consistent with its theoretical monomer mass. M is the molecular weight marker. **B.** HPLC traces of AcrIIIB4 with SAM-AMP and analogues SAH-AMP and SFG-AMP. AcrIIIB4 specifically degrades SAM-AMP into MTA-AMP and HL. The traces of control samples are shown in red and AcrIIIB4 reaction samples in black.

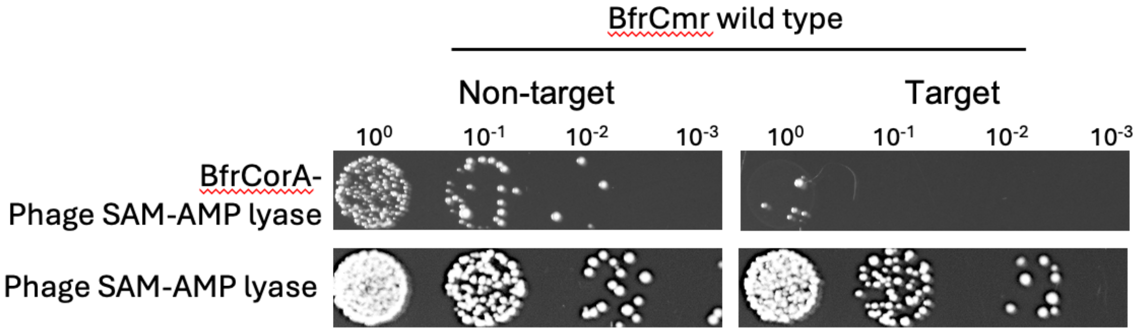

Supplementary Figure 9. Phage encoded SAM-AMP lyase can replace the cellular enzyme *in vivo*. Substituting the bacterial SAM-AMP lyase with the phage counterpart conferred plasmid immunity in the presence of *B. fragilis* Cmr/CorA system, underscoring the essential role of SAM-AMP degradation in mediating immunity and demonstrating that the two proteins have equivalent activities.

Supplementary Table 1. Primers used for SAM-AMP lyase mutagenesis and the synthetic gene encoding AcrIIIB4.

Name	Sequence (5’-3’)	Note
E71Q-Fw	CCGAGAATGGACAAGAAACAGTGGTG	Mutagenesis
E71Q-Rv	CACCACTGTTTCTTGTCCATTCTCGG	Mutagenesis
C66A-Fw	CCAGGAGTGGGGCGCTCCCGAGAATGG	Mutagenesis
C66A-Rv	CCATTCTCGGGAGCGCCCCACTCCTGG	Mutagenesis
Q108A-Fw	GAACCAAATGAAAGCGTCAACGTTAACG	Mutagenesis
Q108A-Rv	CGTTAACGTTGACGCTTTCATTTGGTTC	Mutagenesis
g-block sequence encoding DAN18478.1	GCGCCCATGGCACATATGACCATCCAGGAAAAAATCAAGAAAACGGGGGAGGGT<br>AAATACAAAATTAAATTCTCTACTATCAAACGCTACTCTATCGAGTTTGGACGT<br>ACTGTCACCGATTACTATTTTGTCGAATACAAGAACAACACCTTTATCACAGAA<br>ACACCCTTTATCCTGTTGAAGAACTTCGGCGACAATTTTAGTAAGGACGGTAGC<br>GGCGCGGGTTACTGTTCTGAGATCGTATCATGTGAACGCTACTTCCGCGAAAAT<br>GAATACAAAAATCAGAATTCCCGTACACAGGAGTTTAAGTTAGTCATTGGAATC<br>AACCAGGGGTACAACCATAATAACGATAGTAGTATCAACATCTATGAGTTGTAT<br>CAAGAGGTGGCTGAGAAAATCTATCAGAAATACGGAACGTATATTTCTGCGACA<br>ATTACTGAGAGCAAGGTCATCTATTCGGCGTCATGGGGGTGTCCCGCCGGTGGG<br>GAGATTGTCTACTCTATTAAAGGTACTAAAAACCCTCAGTTTGTAGATGACTTC<br>GAGAAGTACAAAAAAGCCACAGAGCGTGTCGCAAAGACACTGGCAAGTGAACTT<br>AAACAGTCTACTTTCACGCTTACCTGGAAGAATCTGGAGTTGAACTACTTCAAG<br>AAGATCGACGGCTCCTGCAAATGACTCGAGGGATCCCGCG	Protein expression

Supplementary Table 2: Data processing and refinement statistics for apo WT SAM-AMP lyase and the E71Q variant in complex with MTA-AMP

	WT apo SAM-AMP lyase	SAM-AMP lyase E71Q variant with MTA-AMP
**Data processing**		
Space group	P1	P1
Cell dimensions		
a, b, c (Å)	54.4, 55.4, 81.7	53.8, 55.5, 82.0
α, β, χ (°)	75.7, 72.0, 60.8	87.7, 72.6, 61.0
Resolution (Å) (high resolution)	48.0 – 1.70 (1.73 – 1.70)*	46.0 – 1.65 (1.68 – 1.65)*
R_{merge}	0.029 (0.672)*	0.094 (1.369)*
$I/\sigma(I)$	13.9 (1.4)*	10.6 (1.1)*
Completeness (%)	97.6 (96.0)*	97.5 (95.9)*
Average redundancy	3.6 (3.7)	7.2 (6.5)
$CC_{1/2}$	0.99 (0.56)	0.99 (0.63)
V_m (Å^3/Da)	2.38	2.38
Solvent (%)	48.4	48.3
**Refinement**		
Unique reflections	84447 (4431)	92147 (4548)
R_{work} / R_{free}	20.9 / 23.6	18.9 / 22.4
Geometric deviations		
Bonds (Å) / Angles (°)	0.008 / 1.55	0.008 / 1.52
No. atoms (non H)		
Protein	5971	6039
Water	224	514
MTA-AMP	N/A	252
MPD	56	16
B factors (Å^2)		
Protein	42.7	28.7
Water	47.3	36.6
MTA-AMP	N/A	20.2
MPD	43.2	49.0
Ramachandran Favoured / outlier (%)	97.5 / 0	98.2 / 0
Molprobity score / centile (%)	1.5 / 99	0.87 / 100

* Values in parentheses are for the highest-resolution shell.
